# Adrenocortical oncocytoma: 11 Case reports and review of the literature

**DOI:** 10.1097/MD.0000000000008750

**Published:** 2017-12-01

**Authors:** Yazhao Hong, Yuanyuan Hao, Jinghai Hu, Bo Xu, Hongli Shan, Xiaoqing Wang

**Affiliations:** a Department of Urology; b Department of Clinical Laboratory, The First Hospital of Jilin University, Changchun Jilin, P.R. China.

**Keywords:** adrenal tumor, adrenocortical oncocytoma, malignant potential, treatment

## Abstract

**Rationale:**

Adrenocortical oncocytoma is an extremely rare tumor of the adrenal gland. Its diagnostic criteria and biological behavior has not yet reached a consensus. The purpose of this study is to investigate the clinical characteristics of adrenocortical oncocytoma.

**Patient concerns:**

The clinical data from 11 cases of adrenocortical oncocytoma were retrospectively analyzed. Five patients found the tumor incidentally during the healthy examination, and 3 cases found the tumor during the diagnostic work-up for the evaluation of flank pain or hypertension. A female patient manifested virilization, and Cushing's syndrome showed in two patients. The tumor diameter was ranging from 2.0-13.0 cm.

**Diagnoses:**

The serum cortisol, plasma aldosterone and catecholamine metabolites were used to evaluate the function of the tumors, and enhanced CT scan was used to confirm the tumor boundary, enhancement, and lymph nodes condition.

**Interventions:**

Seven cases underwent laparoscopic adrenal tumor resection, 4 patients underwent open surgery. Pathological report indicated adrenocortical oncocytoma in all cases, three of which were potentially malignant.

**Outcomes:**

The patients were followed up for 19-72 months, no local recurrence and distant metastases were detected in 3 cases of malignant potential cases.

**Lessons:**

The majority of adrenocortical oncocytoma with or without function are benign, and close follow-up observation is essential.

## 1 Introduction

Oncocytomas can occur in various organs, notably the kidney, thyroid, pituitary, salivary, parathyroid and lacrimal glands as well as the skin, respiratory, and gastrointestinal tracts. It is composed of oncocytes, which are epithelial cells with acidophilic, granular cytoplasm packed with mitochondria. They are usually benign lesions but have an unpredictable malignant potential.^[1]^

Adrenocortical oncocytoma is an extremely rare tumor of the adrenal gland. It was first reported by Kakimoto^[2]^ in 1986. Since then, no more than 120 cases of adrenocortical oncocytoma have been reported in the English-language literature. Its diagnostic criteria and biological behavior have not yet reached a consensus. Most of oncocytomas are detected occasionally in health check-up, but it is still difficult to identify. Adrenal oncocytoma was descripted as a nonfunctional or functional benign neoplasm with a malignant potential.^[3,4]^ Here, we report 11 patients with adrenal cortical oncocytoma and also make a summary of the clinical and pathological features, as well as diagnostic and treatment points of adrenocortical oncocytoma. This study may have the largest sample size in a single center.

## 2 Case presentations

**Figure 1:**
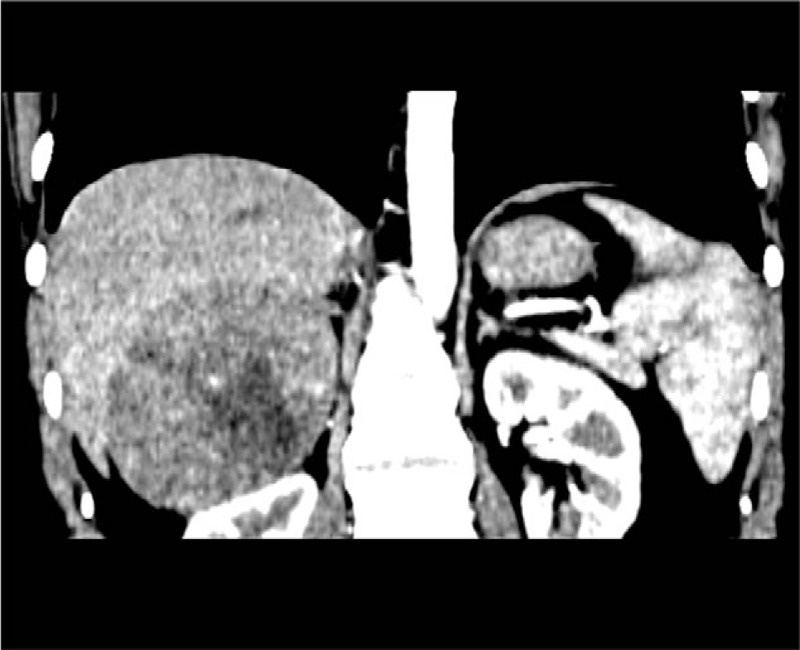
The representative CT scan showed the heterogeneous enhancement of the tumor located between the liver and the right kidney, and necrosis can be seen within the tumor. CT = computed tomography.

**Figure 2:**
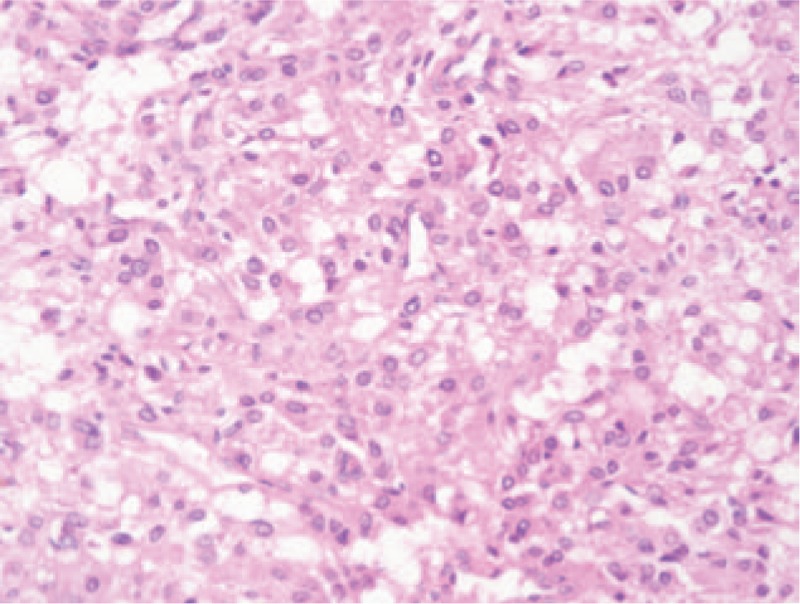
The representative pathological result showed that the tumor cell had the similar size, with acidophil cytoplasm. The cell arranged in the beam-like, and a hollow tubular structure.

The study protocol was approved by the Ethics Committee of the First Hospital of Jilin University. Clinical records of 11 patients with an adrenal oncocytoma, diagnosed and treated at the First Hospital of Jilin University, between January 2008 and January 2012, were retrospectively analyzed. The demographic and clinical data for the patients are listed in Table 1. The group contains 5 males and 6 females; the age range was 6 to 58 years. Five patients found the tumor incidentally during the healthy examination, and 3 cases found the tumor during the diagnostic work-up for the evaluation of flank pain or hypertension. A female patient manifested virilization, such as too much body hair, rough skin, and irregular menstruation; Cushing's syndrome showed in 2 patients with hypertension, rounded face, posterior neck fat deposit, central obesity, and lower abdominal skin purple stripes. The serum cortisol, plasma aldosterone, and catecholamine metabolites were normal except for Cushing Syndrome cases. The CT scan showed that the tumor diameter ranges from 2.0 to 13.0 cm and the CT value is 35 to 48 HU. Enhanced CT showed that 4 lesions can be homogeneously enhanced, whereas the remaining 7 cases are heterogeneous enhancement (Fig. 1). The boundaries between tumor and surrounding tissue were all clear. Seven patients were preoperatively prepared as pheochromocytoma due to the large tumor size, and phenoxybenzamine and blood volume expansion were given. Seven cases underwent laparoscopic adrenal tumor resection, and 4 patients underwent open surgery of adrenal tumors, with tumor diameter ranging from 6.0 to 13 cm. All procedures were successfully performed and no Clavien >2 complications occurred. The tumor specimens were yellow or yellow-brown, encapsulated, and necrosis is common in bulky tumors. Pathological report indicates adrenal cortical oncocytoma in all cases, 3 of which were potentially malignant (Fig. 2). The patients were followed up for 19 to 72 months. The masculine hairy symptoms of the female case disappeared. The symptoms of moon face, buffalo hump also disappeared in 2 cases of the Cushing syndrome cases, at the same time serum cortisol decreased to the normal level. No local recurrence and distant metastases were detected in 3 cases of malignant potential cases.

**Table 1:**
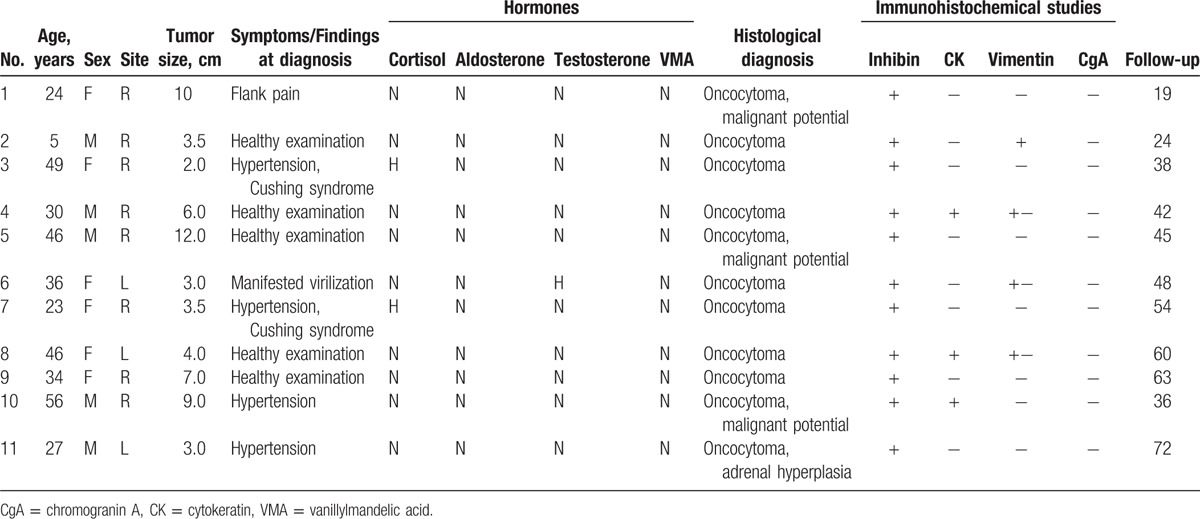
Clinical data of the patients.

## 3 Discussion

Overall incidence of oncocytoma is relatively common in kidney, thyroid, and salivary glands. It can also be found in pituitary, eyelids, parathyroid, thymus, spinal cord, and other parts.^[1,5,6]^ So, the adrenal cortical oncocytoma is a rare disease with an unknown incidence. No identified environmental or genetic risk factor was reported for the etiology of the tumor. It is commonly found in patients between 15 and 77 years. F/M is 2.5/1, and L/R is 3.5/1.^[3,7]^ In the literature, the majority of oncocytoma in the adrenal cortex are nonfunctioning. According to the latest report, 31.5% of the adrenal oncocytoma are related to hormone abnormalities, including hypercortisolism and sex hormone abnormalities.^[8]^ The most common clinical manifestations are Cushing's syndrome, masculine and feminine.^[9]^ Another system review showed that only 17% are functional adrenal masses, and an adrenocortical oncocytic neoplasm occurring with a Cushing's syndrome, pheochromocytoma, or aldosteronoma had been described.^[10]^ In this study, the youngest patient is only 5 years old, and 2.3%(3/11) patients had the functional tumors. According to the clinical complain and laboratory examination, 2 cases were diagnosed as Cushing's syndrome, and 1 case was women masculine. Our results may conclude that adrenocortical oncocytoma may be a functional tumor.

The adrenocortical oncocytoma has no imaging characteristic performance, so it is difficult to confirm the diagnosis preoperatively. The tumor is usually large in size, but has a complete capsule, showing a non invasive growth pattern. In the CT scan, the density is relatively uniform, and the CT value is 20 to 40 HU. In enhanced CT, it shows inhomogeneous enhancement.^[11,12]^ In this study, the tumor diameter ranged from 2 to 13 cm. The CT value was 35 to 48 HU. Enhanced CT showed 4 cases of moderate homogeneous enhancement, and the remaining 7 cases were heterogeneous enhancement.

The management of adrenal mass is surgical removal and the approach depends upon the size and function.^[10]^ Open surgery was the traditional approach for the management of adrenal tumors. With the development of endoscopic techniques, laparoscopic adrenalectomy has become the gold standard for the small adrenal tumors. Compared with open surgery, the laparoscopic surgery has the benefit of less bleeding, faster recovery, and shorter hospital stay. However, for the tumors large than 6 cm and/or of potentially malignancy, because of its high transfer rate and the incidence of complications, laparoscopic adrenalectomy remains controversial.^[13–15]^ In this study, 7 cases underwent laparoscopic adrenal adenoma resection, in which the largest tumor diameter was 8 cm. No serious complications occurred. We think that laparoscopic resection of adrenal tumors is safe. Especially in following circumstances that preoperative CT and MRI results indicate the tumor is encapsulated, the boundaries between tumor and surrounding tissue is clear, no regional lymph nodes invasion.

The diagnosis of adrenocortical oncocytoma is mainly based on histological and immunohistochemistry. Typical oncocytoma is dark brown, and the tumor cells have the same size, with low-fat droplets granular, eosinophilic cytoplasm, with the nuclear in center, arranged in the beam-like hollow tubular structures, occasionally forming microcystis cavity surrounded by slender fibrous septa. A small amount of lymphocytic infiltration can be discovered in the interstitial. Under the electron microscope, in cytoplasm, there are quantities of mitochondria with layered and bubbly ridge, and small electron-dense inclusion bodies. Few oncocytoma has mitotic and necrotic.^[10,16]^ In this study, all cases were pathologically diagnosed as adrenal cortical oncocytoma. Immunohistochemical results showed 3 cases of malignant potential. However, a strong, hormonally active tumor (especially in Cushing's syndrome or in hyperandrogenism) can also have compact eosinophilic cells without lipid vacuoles, and whether such tumors belong to oncocytoma remains controversial.^[10]^ Further work should be done to definite the adrenal oncocytoma in accuracy.

Most of the adrenocortical oncocytomas are benign; only a few tumors were diagnosed as malignant. It is reported that about 22% of patients showed malignant potential.^[17]^ Tumor size and weight are important biological behavior indicators, but these are not accurate indicators. They should be combined with clinical manifestations, biochemical test results, and histological features. Moreover, the Weiss system^[18]^ is a guideline to distinguish benign and malignant neoplasm, but the Weiss system adrenal is not completely reliable. Therefore, Bisceglia et al^[19]^ modified the Weiss system according to the pathological features of adrenal cortex eosinophils tumor as follows: mitosis count> 5/50 HPF, atypical mitosis and venous invasion as the main criteria, and tumor diameter> 10 cm and (or) weight> 200 g, necrosis, capsular invasion, and sinusoids infiltration as the secondary criteria. If it meets any one of the main criteria can be diagnosed as histologically malignant. If it meets one or more secondary criteria, it can be considered as malignant potential. The ones that do not meet any of the main and secondary criteria are benign. In this study, 8 cases were benign because they met neither major criteria nor minor criteria. Although 3 cases were diagnosed malignant potential in condition that they met the secondary criteria, we did not see any metastasis and recurrence during the follow-up. Argyriou et al^[5]^ reported 1 case with a tumor relapse, and it was the only 1 case in the literature. However, the patient had a history of adrenocortical carcinoma, and the diagnosis criteria used by the author aroused a controversy. Therefore, adrenocortical oncocytoma can be treated as a benign tumor. As a result, if the biopsy of an incidental adrenal tumor showed an oncocytoma, the tumor could be safely observed. However, an officially established histological scoring system is needed, and strict follow-up in the future is indispensable.

## 4 Conclusions

The adrenocortical oncocytoma is a rare tumor with or without function. Surgical removal is still the major treatment method. This disease can be treated as a benign tumor. However, molecular studies, precise diagnosis criteria, large clinical samples, and longer follow-up are needed to confirm the results.
